# Beneficial Effects of Poplar Buds on Hyperglycemia, Dyslipidemia, Oxidative Stress, and Inflammation in Streptozotocin-Induced Type-2 Diabetes

**DOI:** 10.1155/2018/7245956

**Published:** 2018-09-18

**Authors:** Shiqin Peng, Ping Wei, Qun Lu, Rui Liu, Yue Ding, Jiuliang Zhang

**Affiliations:** ^1^College of Food Science and Technology, Huazhong Agricultural University, Wuhan 430070, China; ^2^Wuhan Engineering Research Center of Bee Products on Quality and Safety Control, Wuhan 430070, China; ^3^Key Laboratory of Environment Correlative Dietology (Huazhong Agricultural University), Ministry of Education, Wuhan 430070, China

## Abstract

The effects of propolis on blood glucose regulation and the alleviation of various complications caused by diabetes have been widely studied. The main source of propolis in the northern temperate zone is poplar buds. However, there is limited research on the antidiabetic activity of poplar buds. In order to evaluate the effect of poplar buds on type-2 diabetes, crude extract and 50% fraction of poplar buds were used to feed streptozotocin-induced type-2 diabetic mice. The results showed that 50% fraction could increase insulin sensitivity and reduce insulin resistance, as well as decrease the levels of fasting blood glucose, glycated hemoglobin, and glycosylated serum proteins in diabetic mice. Compared with the model control group, the 50% fraction-treated group showed significant decreases of malondialdehyde (MDA) and increases of superoxide dismutase (SOD) in serum and liver homogenate. Moreover, 50% fraction could significantly decrease total cholesterol (TC), alleviate abnormal lipid metabolism, and enhance antioxidant capacity in the serum. For inflammatory factors, feeding of 50% fraction could also reduce the levels of interleukin 6 (IL-6), tumor necrosis factor *α* (TNF-*α*), monocyte chemotactic protein 1 (MCP-1), and cyclooxygenase-2 (COX-2) in liver homogenate. Taken together, our results suggest that crude extract and 50% fraction of poplar buds, particularly the latter, can decrease blood glucose levels and insulin resistance, and 50% fraction can significantly relieve dyslipidemia, oxidative stress, and inflammation caused by type-2 diabetes.

## 1. Introduction

Diabetes mellitus (DM) is a syndrome characterized by chronic hyperglycemia and disorders of fat and protein metabolisms due to an insufficient secretion or impaired action of insulin [1]. Hyperglycemia is considered as a major contributor to the strong oxidative stress in diabetes [2]. The large intake of glucose and macronutrients can lead to obesity. Obesity is the main factor that induces type-2 diabetes (T2DM), and may cause a proinflammatory state accompanied by the increase in oxidative stress, which may interfere with the anti-inflammatory effects of insulin. Besides, due to insulin resistance, hyperglycemia, and oxidative stress caused by T2DM, several diabetic complications may occur, such as nephropathy, retinopathy, neuropathy, cardiovascular diseases, and nonalcoholic hepatitis (NAFLD) [3]. According to the latest Diabetes Map released by the International Diabetes Federation (IDF), there were 425 million diabetics worldwide in 2017, and with the increase in obesity, there has been an obvious increase in the prevalence of diabetes. In addition, there are more than 350 million people with a high risk of diabetes at present [4]. Therefore, it is highly significant to search for natural ingredients with the effect of relieving hyperglycemia caused by T2DM.

Propolis, a kind of gum-like solid material with aromatic odor, is formed from the secretion of the parotid gland and wax gland of bees using substances gathered from the spores and trunks of plants. Generally, raw propolis contains 50% resin [5, 6]. Several studies have demonstrated that propolis has antioxidant [7], antitrioxypurine [8], anti-inflammatory [9], and antidiabetic effects, as well as protective effects to the liver [10]. Flavonoids were shown to have antidiabetic effects through various intracellular signaling pathways [11]. In our previous study, we examined 50% fraction of propolis and 50% fraction of poplar buds by high-performance liquid chromatography (HPLC). It was found that the main components of flavonoids in 50% fraction of poplar buds are quercetin, kaempferol, apigenin, isorhamnetin, chrysin, and galangin, which are similar to the components of 50% fraction of propolis [12]. However, propolis is rare and difficult to collect [9]. Therefore, poplar buds may be an ideal alternative for relieving blood glucose increase and inflammation caused by T2DM.

This study was carried out to evaluate the antidiabetic activities and effects of the crude extract and 50% fraction of poplar buds on diabetes-induced abnormalities in lipid metabolism, oxidative stress, and inflammation.

## 2. Materials and Methods

### 2.1. Materials and Reagents

Poplar buds were collected from Henan, China and identified by Hongcheng Zhang from the Institute of Apicultural Research, Chinese Academy of Agricultural Sciences (Beijing, China). All organic solvents used were of analytical grade and were purchased from Sinopharm Chemical Reagent Co. Ltd. Streptozotocin (STZ) was obtained from Sigma-Aldrich, Inc.

### 2.2. Preparation of Crude Extract and 50% Fraction of Poplar Buds

The powder of poplar buds (40 g) was accurately weighed and mixed with petroleum ether in order to remove the wax. After ultrasonic extraction with 400 mL of 75% ethanol for 1 h, the supernatant was filtered and collected three times. The crude extract of poplar buds (CEPB) was obtained after evaporation and vacuum freeze-drying.

The crude extract of poplar buds was adsorbed by XAD-2 macroporous resin and eluted with 30%, 50%, 80%, and 95% ethanol in turn. The 50% ethanol eluent was collected to prepare the 50% fraction after vacuum concentration and freeze-drying.

### 2.3. Animal Care and Experimental Design

Male Kunming mice (18–20 g) were purchased from the Wuhan Institute of Biological Products Co. Ltd. (certificate of animal quality: SCXK (HuBei) 2012-0013), and kept under environmental conditions of 12 : 12 h light-dark cycle and 22–24°C. High-fat diet was composed of 68.8% normal diet, 15% sugar, 10% lard, 5% egg yolk powder, 1% cholesterol, and 0.2% sodium cholate. After one week of adaptive feeding, the mice were fed with high-fat diet except for the normal control group. After 4 weeks, STZ of 65 mg/kg body weight (BW) was injected intraperitoneally for three consecutive days. After one week, the mice were fasted for 12 h for testing of fasting blood glucose (FBG). Mice with blood glucose levels higher than 7.8 mmol/L in the fasting state were considered as diabetic.

The mice were randomly divided into 6 groups (*n* = 10) as follows: Group 1 (normal control group, NC), normal mice fed with normal chow diet; Group 2 (model group, DM), diabetic mice fed with high-fat diet; Group 3 (metformin group, M), diabetic mice treated with 100 mg/kg BW of metformin and fed with high-fat diet; Group 4 (CEPB group, EPB100), diabetic mice treated with 100 mg/kg BW of CEPB and fed with high-fat diet; Group 5 (50% fraction group, 50%FPB100), diabetic mice treated with 100 mg/kg BW of 50% fraction and fed with high-fat diet; Group 6 (50% fraction group, 50%FPB50), diabetic mice treated with 50 mg/kg BW of 50% fraction and fed with high-fat diet.

The treatments were continued for 4 weeks. STZ was dissolved by citric acid buffer (pH = 4.35). Metformin, CEPB, and 50% fraction were, respectively, mixed twice with PEG6000 and then dissolved by 0.5% CMC-Na. All animals were handled in accordance with the standards for laboratory animals established by the People's Republic of China, the Declaration of Helsinki, and the Guiding Principles in the Care and Use of Animals.

### 2.4. Fasting Blood Glucose (FBG) Determination

FBG was measured every week during the feeding period. The samples were collected from the tail veins and immediately tested by a Roche blood glucose meter (F. Hoffmann-La Roche Ltd., Germany).

### 2.5. Oral Glucose Tolerance Test (OGTT)

One day before the end of the experiment, an oral glucose tolerance test (OGTT) was performed. The mice were fasted for 12 h, and were randomly selected from each group the next morning. Firstly, one drop of blood was taken from the tail tip to determine the fasting blood glucose, and then the mice were fed with 1.0 g/kg BW amylaceum. The blood glucose levels were then determined at 30 min, 60 min, and 120 min after feeding of amylaceum.

### 2.6. Specimen Collection

At the end of the treatments, the mice were fasted for 12 h after the last feeding. Blood samples were taken from the eye after the removal of the eyeball, and then the mice were killed by cervical dislocation. Whole blood samples were centrifuged at 4000 r/min for 10 min at 4°C, and the supernatant was taken as experimental serum. The livers were quickly removed, washed with cold saline, blotted with filter paper, and weighed. Serum and liver were stored at −80°C for subsequent use.

### 2.7. Glycometabolism Determination

The plasma levels of insulin and glycosylated hemoglobin (GHb) were measured by the ELISA kits (Shanghai YuanYe Biotechnology Co. Ltd., Shanghai, China), and the concentration of glycated serum protein (GSP) was determined using commercial kits (Nanjing Jiancheng Biological Engineering Research Center, Nanjing, China) according to the manufacturer's instructions.

### 2.8. Fat Metabolism Determination

The concentrations of total cholesterol (TC), triglyceride (TG), low density lipoprotein cholesterol (LDL-C), and high-density lipoprotein cholesterol (HDL-C) were determined using commercial kits (Nanjing Jiancheng Biological Engineering Research Center, Nanjing, China) according to the manufacturer's instructions.

### 2.9. Oxidative Stress Determination

The concentrations of malondialdehyde (MDA) and superoxide dismutase (SOD) in the serum and liver homogenate were determined using commercial kits (Nanjing Jiancheng Biological Engineering Research Center, Nanjing, China) following the manufacturer's instructions.

### 2.10. Determination of Inflammatory Cytokines

ELISA kits (Shanghai YuanYe Biotechnology Co. Ltd., Shanghai, China) were utilized to measure the levels of interleukin 6 (IL-6), tumor necrosis factor *α* (TNF-*α*), monocyte chemotactic protein 1 (MCP-1), and cyclooxygenase-2 (COX-2) in the liver homogenate.

### 2.11. Analysis and Characterization of 50% Fraction by HPLC-ESI-QTOF-MS/MS

The 50% fraction was analyzed using an Accurate-Mass Q-TOF LC/MS 6520 equipped with a diode array detector (Agilent Technologies, USA). For MS detection, the sample was dissolved in methanol to 1 mg/mL and filtered through a 0.45 *μ*m membrane filter before injection. The column used was the Hypersil GOLD C18 (250 mm × 4.6 mm, 5 *μ*m, Thermo Fisher Scientific Inc., USA). An elution with solvent A (0.3% formic acid) and solvent B (acetonitrile) in a step gradient way at the flow rate of 0.5 mL/min was carried out as follows: 0 min, 15% B; 10 min, 20% B; 15 min, 30% B; 20 min, 35% B; 25 min, 35% B; 30 min, 40% B; 35 min, 45% B; 50 min, 50% B, 70 min, 60% B; 75 min, 90% B; and 80 min, 15% B. The detection wavelength was set at 280 nm. The column temperature was kept at 30°C and the injection volume was 10 *μ*L. ESI conditions were set as follows: negative ion model; dry gas (nitrogen) flow at 10 L/min; dry gas temperature at 350°C; nebulizer pressure at 35 psi; capillary voltage at 3500 V; MS full scan range, m/z 50–1000; and MS/MS 50–1000.

### 2.12. Statistical Analysis

The results were expressed as mean ± standard deviation. The data of the experimental groups were analyzed by SPSS 16.0 with univariate ANOVE analysis and Duncan multiple comparison, and significant effects were examined at a probability level of *p* < 0.05.

## 3. Results

### 3.1. Effects of CEPB and 50% Fraction on FBG

Table 1 demonstrates the changes in FBG after the intake of CEPB and 50% fraction. During the treatment, FBG of diabetic mice firstly increased and then decreased. After 28 days of treatment, there were significant differences in blood glucose levels between the 50% fraction treatment group and model group (*p* < 0.05). 50%FPB100 treatment resulted in less dramatic changes in blood glucose levels compared with other treatments, and showed the effect of reducing blood glucose levels from the first week of administration (*p* < 0.05). Compared with CEPB, 50% fraction showed better effects at the same dose.

### 3.2. Effects of CEPB and 50% Fraction on OGTT

OGTT is a glucose load test that can reflect the function of islet cells in diabetic mice and the ability of the body to regulate blood glucose level, as well as help evaluate the occurrence of impaired glucose tolerance and insulin resistance. After the gavage of amylaceum to normal mice, the blood glucose level would rapidly increase in 15–30 min, and then return to fasting level in 120 min, indicating normal glucose metabolism in the body; however, under the same conditions, the blood glucose level in diabetic mice could hardly be restored to the fasting level after 120 min, implying the abnormal glucose metabolism in these mice [13].

**Figure 1:**
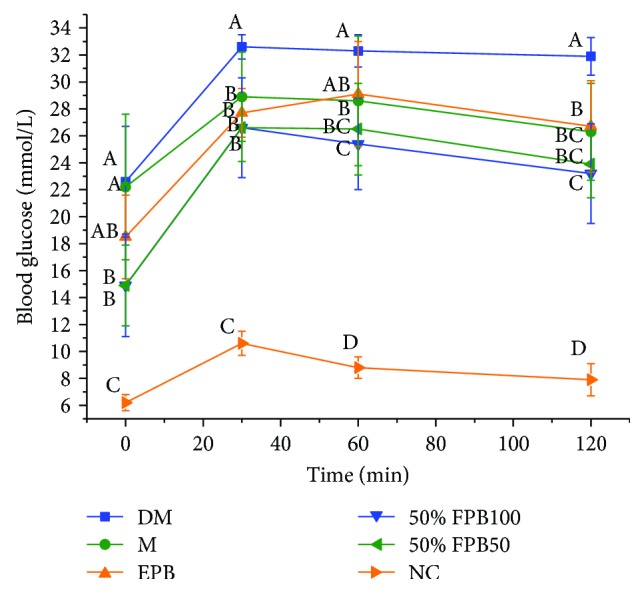
Effects of CEPB and 50% fraction on OGTT in diabetic mice. OGTT: oral glucose tolerance test. In each column, means with different superscript letters differ significantly at *p* < 0.05.

As shown in Figure 1, the blood glucose level in the NC group peaked at 30 min and then returned to the normal level at 120 min. However, diabetic mice displayed increases in blood glucose level at 120 min after glucose administration, especially the DM group. The 50% fraction-treated mice showed a more rapid decrease in blood glucose level than CEPB-treated mice, indicating that 50%FPB100 could better alleviate the abnormalities in glucose metabolism.

### 3.3. Effects of CEPB and 50% Fraction on INS

**Figure 2:**
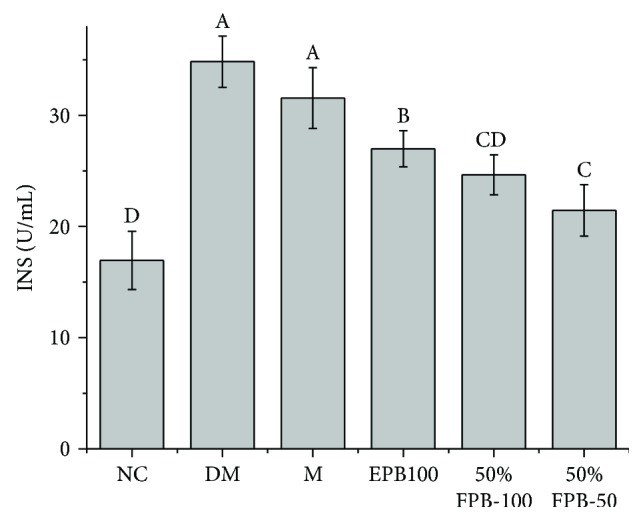
Effects of CEPB and 50% fraction on INS in diabetic mice. INS: insulin. In each column, means with different superscript letters differ significantly at *p* < 0.05.

As can be seen from Figure 2, the secretion of insulin was significantly increased in the DM group (34.83 ± 2.73 mmol/L) compared with that in the NC group (16.93 ± 2.62 mmol/L) (*p* < 0.05), but it was the opposite case for the FBG level (Figure 1), indicating that the experimental mice suffered from T2DM. Compared with the DM group, other groups of diabetic mice all showed significant decreases in the concentrations of insulin in the blood (*p* < 0.05). Based on the results of blood glucose levels, feeding of 50% fraction could better reduce the blood glucose level in diabetic mice and could lead to lower insulin concentration, indicating that 50% fraction can effectively relieve insulin resistance caused by islet *β* cells.

### 3.4. Effects of CEPB and 50% Fraction on GHb and GSP

**Figure 3:**
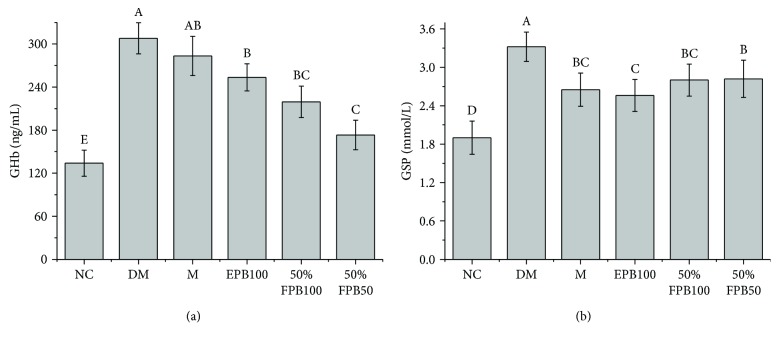
Effects of CEPB and 50% fraction on GHb and GSP in diabetic mice. (a) Content of GHb in serum. (b) Content of GSP in serum. GHb: glycosylated hemoglobin; GSP: glycated serum protein. In each column, means with different superscript letters differ significantly at *p* < 0.05.

Compared with the NC group, the DM group showed significant increases in GSP and GHb levels, indicating that long-term high-fat diet and STZ could induce long-term hyperglycemia symptoms (*p* < 0.05, Figure 3). The gavage of metformin, CEPB, and 50% fraction significantly decreased the levels of GSP and GHb (*p* < 0.05) when compared with the DM group. 50% fraction showed better inhibitory effects on GHb than CEPB (*p* < 0.05). These results suggested that long-term feeding of CEPB and 50% fraction could reduce hyperglycemia in diabetic mice.

### 3.5. Effects of CEPB and 50% Fraction on Fat Metabolism

Elevation of TC is significantly associated with the occurrence of cardiovascular and cerebrovascular diseases in diabetic patients. HDL-C promotes reverse transport of cholesterol, and has anti-inflammatory, antioxidant, and endothelial protective effects, which can reduce the incidence of atherosclerosis. LDL-C is a kind of lipoprotein particle that carry cholesterol into peripheral tissues [14]. Excessive LDL-C can cause the accumulation of cholesterol in arterial walls and increase the incidence of atherosclerosis. TG is often adopted to determine the incidence of coronary heart disease and metabolic syndromes [15]. These indicators are usually measured together to evaluate the lipid metabolism in diabetic patients.

Table 2 shows the effects of treatments by metformin, CEPB, and 50% fraction for 4 weeks on TC, TG, HDL-C, and LDL-C in the serum. The TC, TG, and LDL-C levels in the DM group were significantly higher (*p* < 0.05), and the HDL-C level was significantly lower (*p* < 0.05) compared with the NC group. Compared with the DM group, the 50%FPB100 and 50%FPB50 groups showed significant decreases (*p* < 0.05) in TC and LDL-C values, and the 50%FPB100 group showed a significant increase (*p* < 0.05) in HDL-C value, indicating that 50% fraction could better improve fat metabolism in diabetic mice.

### 3.6. Effects of CEPB and 50% Fraction on SOD and MDA

**Figure 4:**
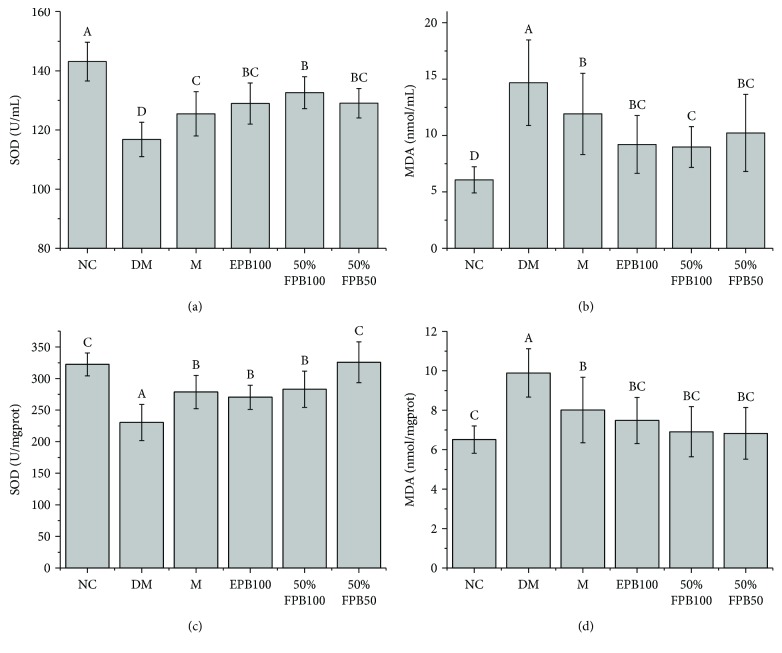
Effects of CEPB and 50% fraction on SOD and MDA in diabetic mice. (a) SOD activity in serum. (b) Content of MDA in serum. (c) SOD activity in the liver homogenate. (d) Content of MDA in the liver homogenate. MDA: malondialdehyde; SOD: superoxide dismutase. In each column, means with different superscript letters differ significantly at *p* < 0.05.

Compared with that of the NC group, the SOD in serum and liver homogenate of the DM group was significantly decreased (*p* < 0.05), while the MDA was significantly increased (*p* < 0.05), indicating the occurrence of severe oxidative stress injury in the diabetic mice (Figure 4). After the treatments, the oxidative stress injury was relieved to varying degrees. Compared with that of metformin-treated mice, the SOD of the 50%FPB100 group significantly increased and the MDA significantly decreased (*p* < 0.05). These results suggested that 50% fraction has a good alleviation effect on the oxidative stress caused by T2DM.

### 3.7. Effects of CEPB and 50% Fraction on Inflammatory Cytokines

**Figure 5:**
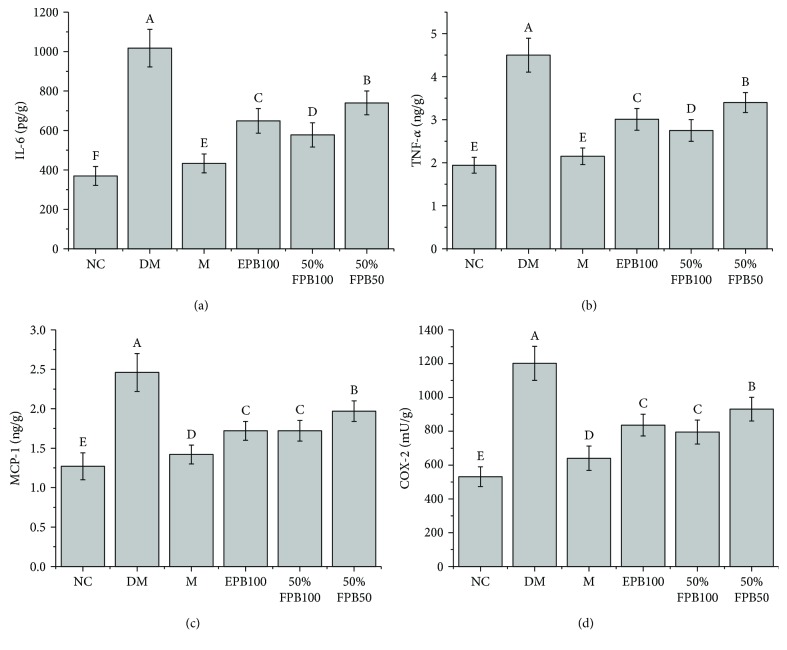
Effects of CEPB and 50% fraction on IL-6, TNF-*α*, MCP-1, and COX-2 in the liver homogenate of diabetic mice. (a) IL-6; (b) TNF-*α*; (c) MCP-1; (d) COX-2. IL-6: interleukin 6; TNF-*α*: tumor necrosis factor *α*; MCP-1: monocyte chemotactic protein 1; COX-2: cyclooxygenase-2. In each column, means with different superscript letters differ significantly at *p* < 0.05.

Mice suffering from T2DM showed higher levels of IL-6, TNF-*α*, MCP-1, and COX-2 in the liver homogenate (Figure 5). We found that both 50% fraction and CEPB could reduce the levels of IL-6, TNF-*α*, MCP-1, and COX-2 production, and a more significant effect was observed for 50% fraction than for CEPB.

### 3.8. Identification of 50% Fraction by HPLC-ESI-QTOF-MS/MS

**Figure 6:**
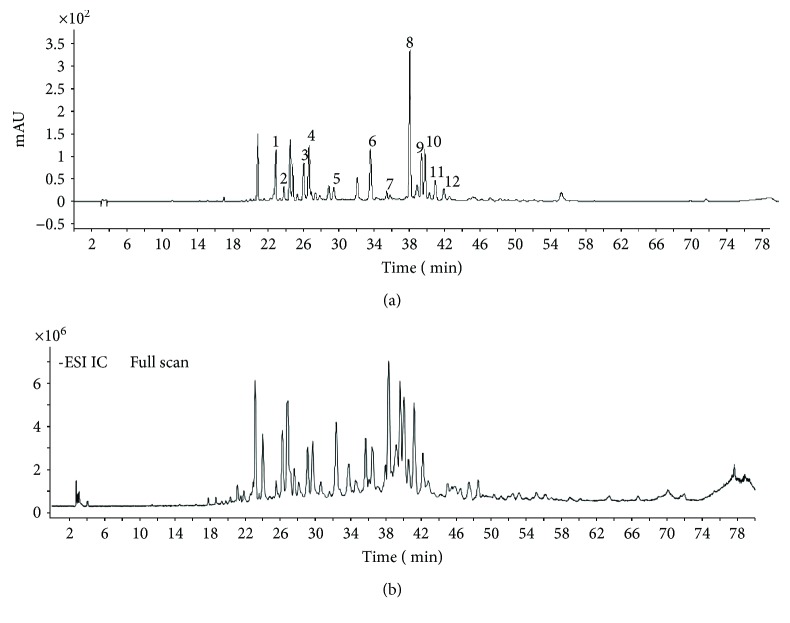
HPLC chromatogram (a) and HPLC-MS total ion chromatogram (b) of 50% fraction.

As shown in Figure 6 and Table 3, 12 kinds of flavonoids were identified by MS, including 5 flavonoids (luteolin, apigenin, chrysin, pinocembrin, and galangin), 4 flavonols (isohamnetin, 3-methoxy quercetin, 5.7-dimethoxy quercetin, and 5-methoxy alpinin), 2 dihydroflavones (pinobanksin and 5-methoxy pinobanksin-3-O-acetate), and 1 dihydroflavonol (3-acetate-pinobanksin).

## 4. Discussion and Conclusion

Previous studies have proposed that T2DM causes autooxidation of glucose, the production of polyalcohols and glycosylation products, dyslipidemia, and low-level inflammation [16–18]. In earlier studies, Croatian propolis [19], Chinese propolis, Brazilian propolis [20], and Egyptian propolis [21] have been found to have ameliorative effects on diabetes. In this work, it can be concluded that poplar buds also have positive effects on diabetes. In addition, 50% fraction could control the diabetes-induced abnormalities in glycometabolism, lipid metabolism, oxidative stress, and inflammation.

A previous study reported that propolis treatment could significantly alleviate the hyperglycemia in STZ-induced diabetic rats [22]. GSP and GHb are the products of long-term nonenzymatic glycosylation of blood glucose and hemoglobin, which can reflect the average blood glucose level during 2–3 weeks and 8–12 weeks, respectively [23]. CEPB and 50% fraction could alleviate the abnormality in GHb and GSP levels to some extent, which is similar to the results of a previous study in which diabetic rats were fed with propolis, indicating that poplar buds can also regulate the blood glucose level of diabetic mice [24].

Dyslipidemia is believed to be a major risk factor for the development of various diabetic complications [25]. Cardiovascular diseases, as one of the main causes of the deterioration and death of patients with T2DM, are mainly related to the changes in plasma lipoproteins, and are often reflected by high levels of TG and LDL-C, and a low level of HDL-C [26]. Our results showed that both 50 mg/kg BW and 100 mg/kg BW of 50% fraction can significantly alleviate the dyslipidemia caused by T2DM.

Insulin resistance is one of the characteristics of T2DM. A previous study has proved that propolis treatment can decrease insulin resistance in obese diabetic rats [27]. In this study, the insulin level in the 50%FPB100 group declined significantly (*p* < 0.05) after 4 weeks of feeding, and the abnormalities in hyperglycemia and dyslipidemia were alleviated to some extent. Currently, we still do not have a reasonable explanation for the slight differences between the 50%FPB50 and 50%FPB100 groups.

It has been confirmed that there is a close link between oxidative stress and insulin resistance [28]. Inhibition of oxidative stress could be a potential approach to treat T2DM [29]. Increases in free radical level could initiate a decrease in insulin secretion in response to hyperglycemia, which could result in more serious hyperglycemia and diabetes [30]. The results of MDA and SOD levels showed that 50% fraction could significantly reduce the oxidative injury caused by T2DM.

In addition, a previous study has demonstrated the relationship between diabetes and inflammation [31]. It is well known that diabetes causes the occurrence of hyperglycemia, insulin resistance, and damage of *β* cells [32]. Dyslipidemia and oxidative stress also contribute significantly to the low-level inflammation [33, 34]. IL-6, COX-2, TNF-*α*, and MCP-1 are closely related to the inflammatory process [35]. IL-6 is an important proinflammatory cytokine that can aggravate inflammation to cause tissue damage [36]. As an important factor in a cytokine network, TNF-*α* could promote the generation of other inflammatory cytokines, and at the same time cause physiological and pathological changes in the body [36, 37]. COX-2 is a rate limiting enzyme involved in the synthesis of prostaglandin E2, and they collectively enhance inflammatory responses [38]. A macrophage with high proliferative activity could produce MCP-1, which would elevate the chronic inflammation in the body due to the activation of hyaline leukocyte and macrophage [39]. In this study, we observed that IL-6, COX-2, TNF-*α*, and MCP-1 in the liver homogenate of diabetic mice significantly decreased after the treatment of 50% fraction, which is in agreement with the results of other studies that reported the anti-inflammatory properties of propolis [40–42].

In conclusion, this study demonstrated that poplar buds are effective in ameliorating the abnormalities in glycometabolism, dyslipidemia, and inflammation caused by T2DM. However, it remains unclear what specific active ingredients in 50% fraction are responsible for the antidiabetic activity. Besides, more evidences are required to explain why a low dosage of 50% fraction was better than a high dosage of 50% fraction in regulating INS and GHb. Therefore, our future research will be focused on the specific active ingredients and appropriate dose of 50% fraction for regulating the glucose level in T2DM.

## Figures and Tables

**Table 1:** Effects of CEPB and 50% fraction on FBG in diabetic mice.

Group	FBG (mmol/L)				
	0 d	7 d	14 d	21 d	28 d
NC	5.4 ± 1.0^b^	5.4 ± 0.8^c^	6.6 ± 1.3^c^	6.1 ± 0.9^c^	6.4 ± 0.7^c^
DM	12.4 ± 3.8^a^	20.2 ± 5.7^a^	26.4 ± 3.9^a^	24.7 ± 5.3^a^	21.1 ± 4.3^a^
M	11.7 ± 2.7^a^	15.2 ± 6.3^ab^	12.8 ± 6.9^b^	16.1 ± 4.5^b^	19.3 ± 6.7^ab^
EPB100	10.4 ± 2.6^a^	19.2 ± 4.6^ab^	25.5 ± 4.3^ab^	17.3 ± 4.0^b^	19.6 ± 6.6^ab^
50%FPB100	11.3 ± 3.8^a^	14.6 ± 5.2^b^	18.2 ± 5.3^b^	15.3 ± 4.7^b^	15.7 ± 4.2^b^
50%FPB50	11.9 ± 3.5^a^	19.4 ± 7.7^ab^	20.1 ± 6.2^b^	16.9 ± 5.3^b^	16.2 ± 3.8^b^

In each column, means with different superscript letters differ significantly at *p* < 0.05.

**Table 2:** Effects of CEPB and 50% fraction on TC, TG, HDL-C, and LDL-C in diabetic mice.

Group	TC (mmol/L)	TG (mmol/L)	HDL-C (mmol/L)	LDL-C (mmol/L)
NC	5.28 ± 1.17^d^	0.76 ± 0.27^b^	2.81 ± 0.60^a^	1.89 ± 0.48^c^
DM	11.26 ± 1.14^a^	1.03 ± 0.34^a^	1.41 ± 0.39^c^	5.02 ± 0.78^a^
M	9.20 ± 1.16^b^	1.06 ± 0.20^a^	1.74 ± 0.39^bc^	4.13 ± 0.86^b^
EPB100	8.02 ± 2.06^bc^	1.16 ± 0.34^a^	1.74 ± 0.32^bc^	4.40 ± 0.67^ab^
50%FPB100	7.70 ± 1.81^c^	1.06 ± 0.28^a^	1.88 ± 0.57^b^	3.78 ± 0.93^b^
50%FPB50	8.45 ± 1.62^bc^	1.01 ± 0.24^a^	1.82 ± 0.52^bc^	4.14 ± 0.48^b^

TC: total cholesterol; TG: triglyceride; HDL-C: high-density lipoprotein cholesterol; LDL-C: low-density lipoprotein cholesterol. In each column, means with different superscript letters differ significantly at *p* < 0.05.

**Table 3:** MS information and UV absorption from the identified compounds of 50% fraction.

Peak number	*R*_*t*_ (min)	UV *λ*_ma*x*_ (nm)	MS (*M* − *H*)−	MS^2^	Compound name	Specie
1	23.08	253, 348	285.07	241.08, 179.04	Luteolin	Flavonoids
2	24.07	253, 364	315.04	300.02	Isorhamnetin	Flavonols
3	26.26	337	269.04	225.05, 181.06, 151.00	Apigenin	Flavonoids
4	26.93	280	271.06	197.59,125.02	Pinobanksin	Dihydroflavone
5	29.73	300	329.06	268.61, 240.05, 177.01	5-Methoxy pinobanksin-3-O-acetate	Dihydroflavone
6	33.76	311	315.05	299.82	3-Methoxy quercetin	Flavonols
7	35.72	/	329.06	314.04	5.7-Dimethoxy quercetin	Flavonols
8	38.33	267, 313	253.05	209.06, 143.05, 107.02	Chrysin	Flavonoids
9	39.61	289	255.06	213.05, 150.99, 107.01	Pinocembrin	Flavonoids
10	40.07	265, 310	269.04	169.06, 197.04, 212.39	Galangin	Flavonoids
11	41.18	294	313.07	253.05, 271.09	3-Acetate-pinobanksin	Dihydroflavonol
12	42.20	265	283.06	268.03, 239.03, 211.04	5-Methoxy alpinin	Flavonols

## Data Availability

The data used to support the findings of this study are available from the corresponding author upon request.

## References

[1] Samadi N., Mozaffari-Khosravi H., Rahmanian M., Askarishahi M. (2017). Effects of bee propolis supplementation on glycemic control, lipid profile and insulin resistance indices in patients with type 2 diabetes: a randomized, double-blind clinical trial. *Journal of Integrative Medicine*.

[2] Al-Assaf A. H. (2012). Antihyperglycemic and antioxidant effect of ginger extract on streptozotocin-diabetic rats. *Pakistan Journal of Nutrition*.

[3] Forbes J. M., Cooper M. E. (2013). Mechanisms of diabetic complications. *Physiological Reviews*.

[4] International Diabetes Federation *IDF Diabetes Atlas*.

[5] Burdock G. A. (1998). Review of the biological properties and toxicity of bee propolis (propolis). *Food and Chemical Toxicology*.

[6] Pietta P. G., Gardana C., Pietta A. M. (2002). Analytical methods for quality control of propolis. *Fitoterapia*.

[7] Dudonné S., Poupard P., Coutière P. (2011). Phenolic composition and antioxidant properties of poplar bud (*Populus nigra*) extract: individual antioxidant contribution of phenolics and transcriptional effect on skin aging. *Journal of Agricultural and Food Chemistry*.

[8] Havlik J., Rada V., Plachy V. (2011). Xanthine oxidase-inhibitory and hypouricemic action of black poplar bud extract. *Planta Medica*.

[9] Wang K., Zhang J., Ping S. (2014). Anti-inflammatory effects of ethanol extracts of Chinese propolis and buds from poplar (*Populus* × *canadensis*). *Journal of Ethnopharmacology*.

[10] De Marco S., Piccioni M., Pagiotti R., Pietrella D. (2017). Antibiofilm and antioxidant activity of propolis and bud poplar resins versus *Pseudomonas aeruginosa*. *Evidence-based Complementary and Alternative Medicine*.

[11] Babu P. V. A., Liu D., Gilbert E. R. (2013). Recent advances in understanding the anti-diabetic actions of dietary flavonoids. *The Journal of Nutritional Biochemistry*.

[12] Ping W., Yue D., Qun L., Jun T., Jiuliang Z., Rui L. (2018). Flavonoids in propolis and poplar resin and their inhibition of *α*-glucosidase activity. *Journal of Huazhong Agricultural University*.

[13] Rozman J., Rathkolb B., Neschen S. (2011). Glucose tolerance tests for systematic screening of glucose homeostasis in mice. *Current Protocols in Mouse Biology*.

[14] Abdul Sani N. F., Belani L. K., Pui Sin C. (2014). Effect of the combination of gelam honey and ginger on oxidative stress and metabolic profile in streptozotocin-induced diabetic Sprague-Dawley rats. *BioMed Research International*.

[15] Kherroubi M., Buysschaert M., Pollak F. (2007). Dyslipidemia and type 2 diabetes mellitus. *Louvain Médical*.

[16] Fu M. X., Requena J. R., Jenkins A. J., Lyons T. J., Baynes J. W., Thorpe S. R. (1996). The advanced glycation end product, Nepsilon-(carboxymethyl)lysine, is a product of both lipid peroxidation and glycoxidation reactions. *The Journal of Biological Chemistry*.

[17] Maritim A. C., Sanders R. A., Watkins J. B. (2003). Diabetes, oxidative stress, and antioxidants: a review. *Journal of Biochemical and Molecular Toxicology*.

[18] Thornalley P. J., Langborg A., Minhas H. S. (1999). Formation of glyoxal, methylglyoxal and 3-deoxyglucosone in the glycation of proteins by glucose. *The Biochemical Journal*.

[19] Oršolić N., Sirovina D., Končić M. Z., Lacković G., Gregorović G. (2012). Effect of Croatian propolis on diabetic nephropathy and liver toxicity in mice. *BMC Complementary and Alternative Medicine*.

[20] Zhu W., Li Y.-H., Chen M.-L., Hu F.-L. (2010). Protective effects of Chinese and Brazilian propolis treatment against hepatorenal lesion in diabetic rats. *Human & Experimental Toxicology*.

[21] El-Sayed E.-S. M., Abo-Salem O. M., Aly H. A., Mansour A. M. (2009). Potential antidiabetic and hypolipidemic effects of propolis extract in streptozotocin-induced diabetic rats. *Pakistan Journal of Pharmaceutical Sciences*.

[22] Thulesen J., Orskov C., Holst J. J., Poulsen S. S. (1997). Short-term insulin treatment prevents the diabetogenic action of streptozotocin in rats. *Endocrinology*.

[23] Cruickshank J. K. (2010). Survival as a function of HbA1c in people with type 2 diabetes. *The Lancet*.

[24] Oladayo M. I. (2016). Nigerian propolis improves blood glucose, glycated hemoglobin A1c, very low-density lipoprotein, and high-density lipoprotein levels in rat models of diabetes. *Journal of Intercultural Ethnopharmacology*.

[25] Mooradian A. D. (2009). Dyslipidemia in type 2 diabetes mellitus. *Nature Clinical Practice. Endocrinology & Metabolism*.

[26] Taskinen M. R., Boren J. (2015). New insights into the pathophysiology of dyslipidemia in type 2 diabetes. *Atherosclerosis*.

[27] Aoi W., Hosogi S., Niisato N. (2013). Improvement of insulin resistance, blood pressure and interstitial pH in early developmental stage of insulin resistance in OLETF rats by intake of propolis extracts. *Biochemical and Biophysical Research Communications*.

[28] Martin B. C., Warram J. H., Krolewski A. S. (1992). Role of glucose and insulin resistance in development of type 2 diabetes mellitus: results of a 25-year follow-up study. *The Lancet*.

[29] Elissa L. A., Elsherbiny N. M., Magmomah A. O. (2015). Propolis restored adiponectin level in type 2 diabetes through PPARγ activation. *Egyptian Journal of Basic and Applied Sciences*.

[30] Zhao L., Pu L., Wei J. (2016). Brazilian green propolis improves antioxidant function in patients with type 2 diabetes mellitus. *International Journal of Environmental Research and Public Health*.

[31] Domingueti C. P., Dusse L. M. S.'. A., Carvalho M. d. G., de Sousa L. P., Gomes K. B., Fernandes A. P. (2016). Diabetes mellitus: the linkage between oxidative stress, inflammation, hypercoagulability and vascular complications. *Journal of Diabetes and its Complications*.

[32] Wellen K. E., Hotamisligil G. S. (2005). Inflammation, stress, and diabetes. *Journal of Clinical Investigation*.

[33] Aouacheri O., Saka S., Krim M., Messaadia A., Maidi I. (2015). The investigation of the oxidative stress-related parameters in type 2 diabetes mellitus. *Canadian Journal of Diabetes*.

[34] Ståhlman M., Fagerberg B., Adiels M. (2013). Dyslipidemia, but not hyperglycemia and insulin resistance, is associated with marked alterations in the HDL lipidome in type 2 diabetic subjects in the DIWA cohort: impact on small HDL particles. *Biochimica et Biophysica Acta (BBA) - Molecular and Cell Biology of Lipids*.

[35] Margetic S. (2012). Inflammation and haemostasis. *Biochemia Medica*.

[36] Scheller J., Chalaris A., Schmidt-Arras D., Rose-John S. (2011). The pro- and anti-inflammatory properties of the cytokine interleukin-6. *Biochimica et Biophysica Acta (BBA) - Molecular Cell Research*.

[37] Tilg H., Trehu E., Atkins M. B., Dinarello C. A., Mier J. W. (1994). Interleukin-6 (IL-6) as an anti-inflammatory cytokine: induction of circulating IL-1 receptor antagonist and soluble tumor necrosis factor receptor p55. *Blood*.

[38] Remppis A., Bea F., Greten H. J. (2010). *Rhizoma coptidis* inhibits LPS-induced MCP-1/CCL2 production in murine macrophages via an AP-1 and NF*κ*B-dependent pathway. *Mediators of Inflammation*.

[39] Dihingia A., Ozah D., Baruah P. K., Kalita J., Manna P. (2018). Prophylactic role of vitamin K supplementation on vascular inflammation in type 2 diabetes by regulating the NF-*κ*B/Nrf2 pathway via activating Gla proteins. *Food & Function*.

[40] Martin L. F. T., Rocha E. M., Garcia S. B., Paula J. S. (2013). Topical Brazilian propolis improves corneal wound healing and inflammation in rats following alkali burns. *BMC Complementary and Alternative Medicine*.

[41] El Rabey H. A., Al-Seeni M. N., Bakhashwain A. S. (2017). The antidiabetic activity of *Nigella sativa* and propolis on streptozotocin-induced diabetes and diabetic nephropathy in male rats. *Evidence-based Complementary and Alternative Medicine*.

[42] Huang J., Wang X., Tao G. (2018). Feruloylated oligosaccharides from maize bran alleviate the symptoms of diabetes in streptozotocin-induced type 2 diabetic rats. *Food & Function*.

